# Brucellosis-induced peritonitis and abdominal aortitis in a non-endemic area patient on peritoneal dialysis: a case report and literature review

**DOI:** 10.3389/fmed.2024.1393548

**Published:** 2024-04-18

**Authors:** Yiqi Huang, Xingyu Zhu, Weigang Shen, Yueping Wang, Meixiang Han

**Affiliations:** ^1^Department of Nephrology, Shaoxing Second Hospital, Shaoxing, Zhejiang, China; ^2^Department of Nephrology, Hangzhou Hospital of Traditional Chinese Medicine, Hangzhou Traditional Chinese Medicine Hospital Affiliated to Zhejiang Chinese Medical University, Hangzhou, Zhejiang, China

**Keywords:** brucella, peritoneal dialysis, peritonitis, abdominal aortitis, case report

## Abstract

**Background:**

Brucella infection is uncommon among peritoneal dialysis (PD) patients in non-endemic areas, and the occurrence of both peritonitis and abdominal aortitis is rare.

**Case presentation:**

In December 2023, a 63-year-old male patient undergoing PD was admitted to Shaoxing Second Hospital due to fever, abdominal pain, and cloudy dialysate. Upon physical examination, diffuse mild abdominal pain and tenderness were observed. Subsequent investigation into the patient's medical history revealed consumption of freshly slaughtered lamb from local farmers 3 days prior to the onset of symptoms. Various diagnostic tests, including routine blood tests, procalcitonin levels, and PD fluid analysis, indicated the presence of infection. Abdominal computed tomography (CT) imaging revealed localized lumen widening of the abdominal aorta with surrounding exudative changes. On the sixth day in the hospital, blood and PD fluid cultures confirmed *Brucella melitensis* infection. The patient was diagnosed with brucella-associated peritonitis and aortitis. Treatment was adjusted to include rifampin and doxycycline for 6 weeks, and the decision was made to keep the PD catheter. Remarkably, the patient exhibited resolution of peritonitis and abdominal aortitis within the initial week of the adjusted treatment. Currently, the patient continues to receive ongoing clinical monitoring.

**Conclusion:**

Brucella is rare but can cause PD-associated peritonitis and arteritis. Prompt diagnosis and treatment can lead to a good outcome in PD patients. Dual therapy is effective, but the need for catheter removal is unclear. Consider international guidelines and patient factors when deciding on catheter removal.

## 1 Introduction

Brucellosis, a zoonotic disease, is often spread through contact with animals or contaminated food, and is frequently missed (1). Research indicates that an estimated 500,000 cases of brucellosis are diagnosed each year on a global scale, with the preponderance of instances concentrated in Asia, Africa, and the Americas (2). Notably, nations within the Eastern Mediterranean, such as Syria, Turkey, and Iraq, demonstrate the highest prevalence rates, ranging from 0.029 to 200.41 cases per 100,000 individuals (3). Furthermore, certain Asian countries have observed a marked uptick in diagnoses in recent years, contrasting with a decrease in annual incidence rates in Europe and the Americas (3).

The clinical manifestation of brucellosis often presents with a diverse array of non-specific symptoms, such as fever, fatigue, hepatosplenomegaly, and involvement of multiple organ systems including the skeletal system (50%), central nervous system (10%), and reproductive system (2–20%) (4). It is important to highlight that abdominal involvement is a rare occurrence in cases of brucellosis infection.

Peritonitis is the main complication in peritoneal dialysis (PD) patients (5), leading to dialysis failure and necessitate catheter removal in up to 20% of cases (6). To the best of our knowledge, only ten cases of brucellosis peritonitis have been reported in these patients, with no other sites affected. In this study, we report a case of PD complicated by brucella-induced peritonitis and abdominal aortitis in a non-endemic area of China. Additionally, we offer a comprehensive review of the clinical manifestations, diagnostic methods, and therapeutic interventions for this uncommon condition as documented in the existing literature.

## 2 Case presentation

On November 13, 2023, a 56-year-old male was admitted to the Nephrology Department of Shaoxing Scecond Hospital (Shaoxing, China) for a 1-day history of fever and abdominal pain, along with cloudy dialysate. Upon further investigation, he disclosed buying fresh mutton at a local private farm and consuming mutton hotpot at home 3 days before the onset of symptoms. The individual, who has undergone PD for a decade as a result of end-stage renal disease stemming from IgA nephropathy, encountered two instances of peritonitis, the most recent of which took place half a year ago. He consistently follows the conventional Continuous Ambulatory Peritoneal Dialysis (CAPD) protocol, performing four exchanges of 2,000 ml of 1.5% PD solution per day.

**Figure 1:**
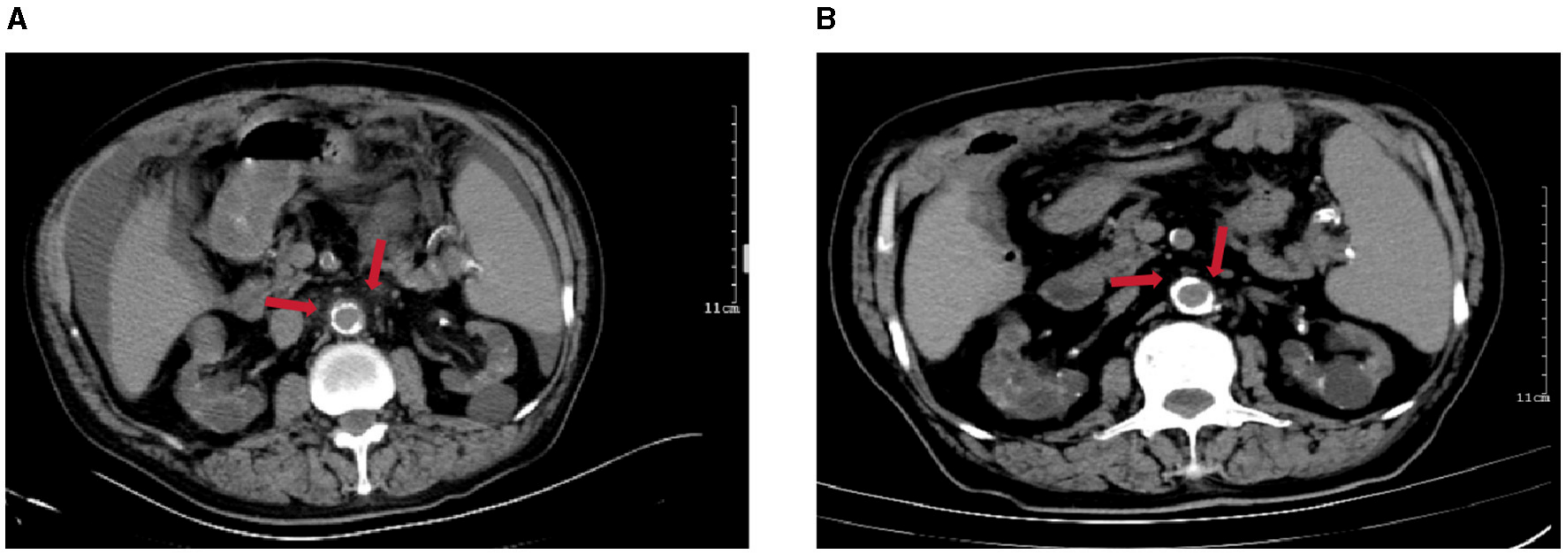
Abdominal CT imaging. **(A)** CT on admission showed a widening of the local lumen of the abdominal aorta and a change in peripheral exudation (arrows), suggesting abdominal aortitis; **(B)** CT on the recovery phase (after taking rifampicin and doxycycline orally for 1 week) showed the inflammation around the abdominal aorta had well-absorbed (arrows).

Upon admission, the physical examination revealed blood pressure of 155/83 mmHg, body temperature of 38.9°C, diffuse abdominal moderate tenderness without rebound pain or muscle tension. Laboratory examination was as follows: white blood cell (WBC) count of 19.3 × 109 cells/L), neutrophils 83.1%, hemoglobin 8.9 g/dL, platelet count of 173 × 109/L, C-reactive protein (CRP) 167.5 mg/L, procalcitonin (PCT) 61 ng/ml, albumin 28 g/L. The PD fluid appeared turbid with a pale yellow hue, and had a total WBC count of 22,136/mm^3^, with 95.7% neutrophils. Computed tomography (CT) scan of the abdomen showed a localized dilation and multiple exudative changes of the abdominal aorta (Figure 1). Based on the patient's clinical data, empiric treatment with piperacillin-tazobactam (intravenous, 4.5 g/dose, twice a day) and vancomycin (intraperitoneal, 1 g/dose, first day; 0.5 g/dose, second and third days) was initiated for a presumptive diagnosis of PD-associated peritonitis and abdominal aortitis.

**Figure 2:**
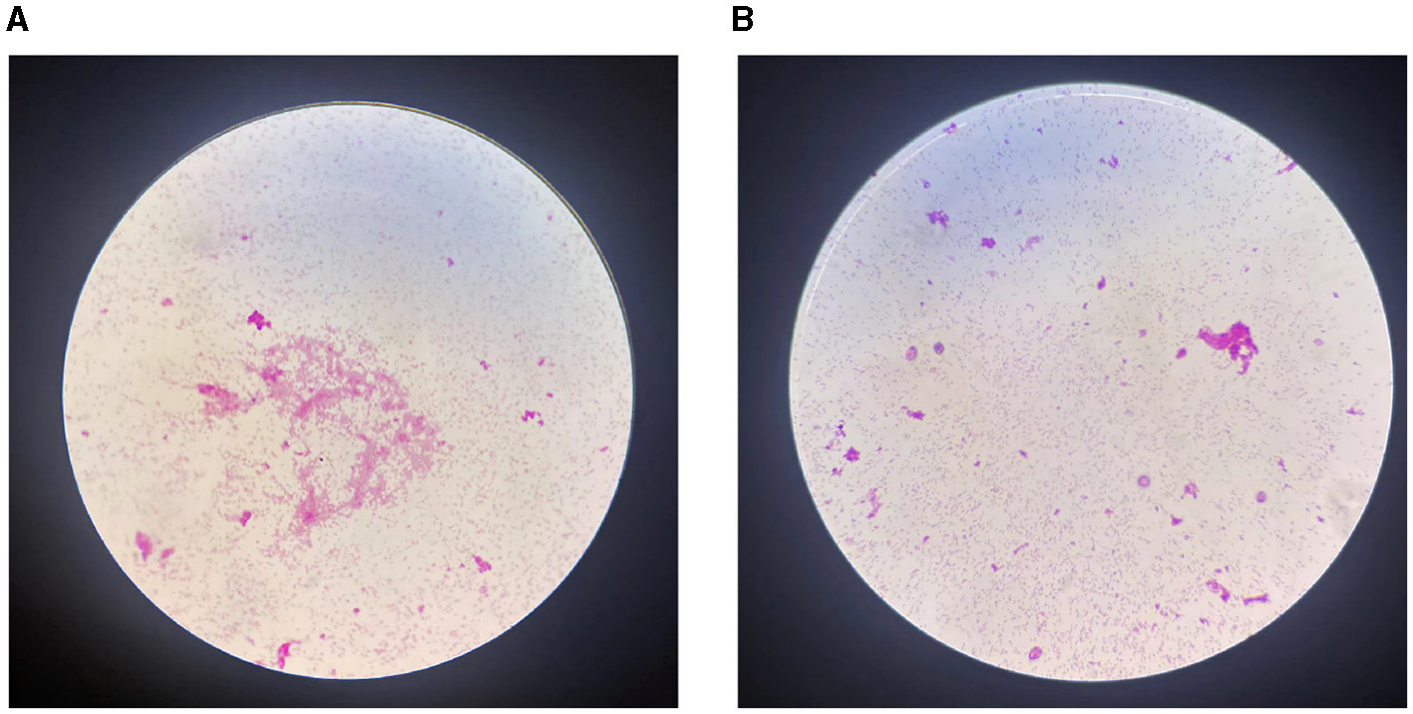
Microbiological examination results. **(A)** Gram staining suggested gram-negative bacilli in the blood culture bottle (magnification, ×1,000); **(B)** Gram staining suggested gram-negative bacilli in the dialysate culture bottle (magnification, ×1,000).

**Figure 3:**
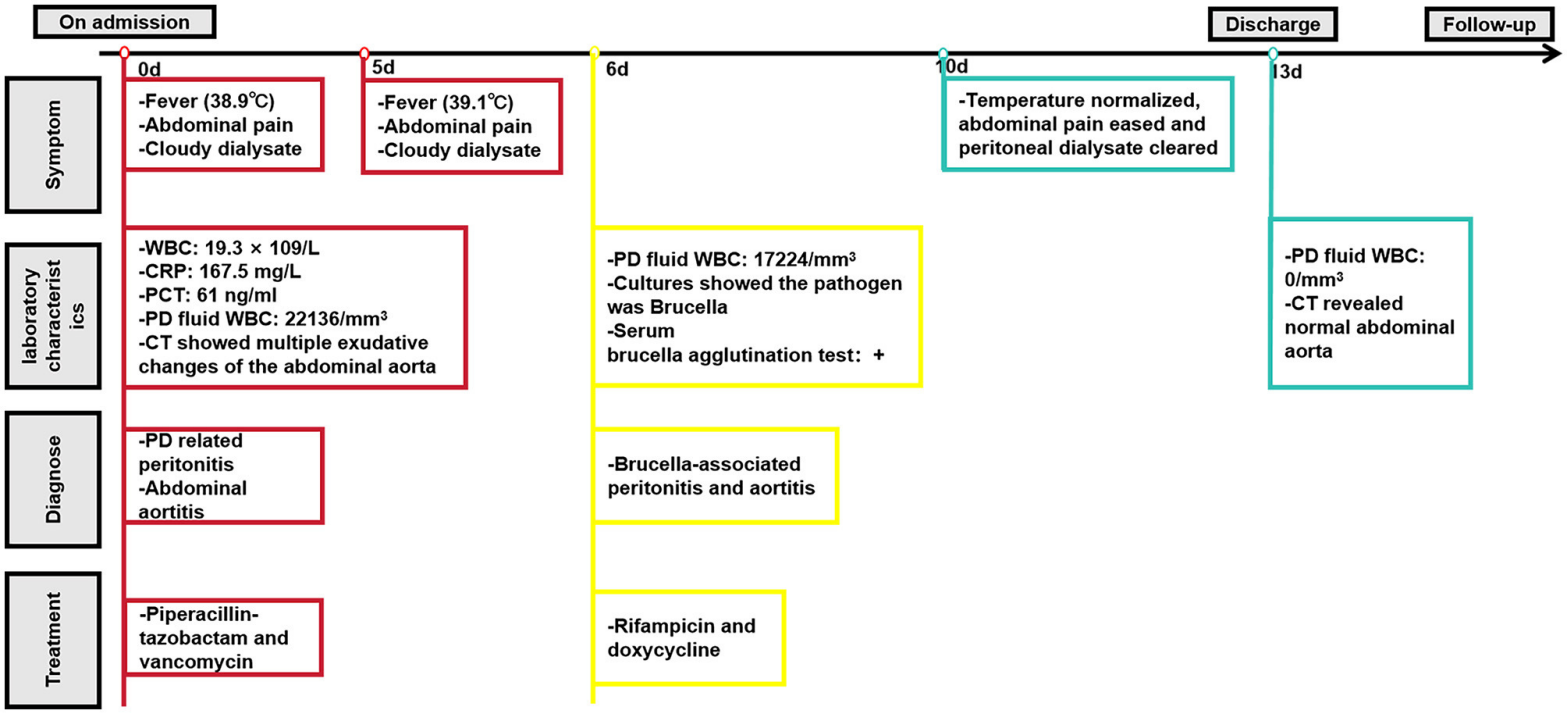
The timeline for diagnosis and treatment.

However, after a period of 5 days, there was no improvement in the patient's clinical status, and analysis of multiple peritoneal dialysate samples revealed a WBC count exceeding 10,000/mm^3^, indicating potential ineffectiveness of empirical anti-infective treatment. On the fifth day of hospitalization, the automated blood culture system detected bacterial growth in the aerobicblood culture bottle and prompting transfer to a Columbia blood plate. Following a 24-h incubation period, *Brucella melitensis* was identified through automated microbiologic analysis (Figure 2). Meanwhile, *Brucella melitensis* was also isolated from the dialysate culture. Additionally, serum brucella agglutination test was markedly positive (titer: 1:1,000). The final diagnosis of were established 7 days after the patient were admitted to the hospital. Finally, the patient was diagnosed with brucella-associated peritonitis and aortitis, and treatment was adjusted to rifampicin (600 mg/dose, once a day) and doxycycline (100 mg/dose, twice a day) for 6 weeks. We suggest using the standard doses of oral doxycycline and rifampicin for PD patients, as the dosages of these medications do not require modification based on glomerular filtration rate. Furthermore, the decision not to remove the PD catheter was made based on the patient's strong preference to continue with CAPD and the patient's geographical distance from the hemodialysis center. On the 4th day following the modification of the treatment regimen, the patient's temperature normalized, abdominal pain eased, and peritoneal dialysate cleared. By the 7th day post-treatment adjustment, the WBC count in the peritoneal dialysate was recorded as 0 and abdominal CT revealed normal abdominal aorta (Figure 1). Now the patient is currently under ongoing clinical observation. Figure 3 illustrates the timeline for diagnosis and treatment.

## 3 Discussion and conclusion

Brucella, a kind of Gram-negative bacterium lacking capsular structure and non-motile, demonstrates the ability to persist in various domestic animal species including cattle, sheep, and pigs. Among these species, *Brucella melitensis* exhibits the highest epidemic potential and pathogenicity. Brucellosis, a zoonotic systemic infectious disease caused by Brucella, is prevalent worldwide, particularly in developing nations and regions with intensive agricultural practices (2). Individuals with a heightened susceptibility to brucellosis encompass veterinarians, farm workers, dairy cattle handlers, and other professions involved in direct contact with Brucella-infected animals. Furthermore, the consumption of inadequately processed animal products, such as undercooked milk, cheese, and meat, poses a risk of Brucella infection to humans (7). The clinical presentation of brucellosis is characterized by non-specific and multi-systemic manifestations (8–10). During the acute phase, patients may experience fever, headache, muscle and joint pain, fatigue, hepatosplenomegaly, lymphadenopathy, and other non-specific symptoms (11). Without appropriate treatment, individuals may progress to a chronic phase marked by recurrent fever, arthritis, and chronic fatigue. Additionally, brucellosis can lead to various complications, with lymphadenopathy, bone marrow abscess, and epididymitis being among the most common (9).

Peritonitis, a rare complication of brucellosis, exhibits a notably higher incidence in PD patients compared to the general population (12). This elevated risk is believed to stem from prolonged PD, which can lead to increased peritoneal permeability and fibrosis (13), ultimately compromising defense mechanisms. The literature currently reports only 10 cases of brucellosis-related peritonitis in PD patients, with the majority of cases originating from Turkey and Saudi Arabia (10, 14–20), and a single case from China (21). Table 1 offers a comprehensive summary of these cases.

**Table 1:** Case summary of Brucella infection in PD patients.

**References**	**Country**	**Gender**	**Age**	**Previous peritonitis**	**Infectious source**	**Clinical presentation**	**Culture**	**Infection part**	**Treatment**	**Outcome**
Al et al. (14)	Saudi Arabia	Female	14	Yes	Unknown	Abdominal pain	Peritoneal fluid culture (+)	Enterocoelia	Rifampicin and doxycycline for 6 weeks	Continued PD
Niu et al. (21)	China	Female	54	Yes	Unpasteurized beef consumption	Abdominal pain, cloudy PD effluent and ultrafiltration decrease	Peritoneal fluid culture (+)	Enterocoelia	Rifampicin, minocycline, and levofloxacin for 18 weeks	Continued PD
Muhammad (15)	Saudi Arabia	Male	45	Unknown	Unpasteurized cheese consumption	Fever, generalized dull abdominal pain, vomiting and diarrhea	Peritoneal fluid culture (+)	Enterocoelia	Minocycline and ciprofloxacin for 12 weeks	PD catheter removed and shift to hemodialysis
Koz et al. (16)	Turkey	Male	49	Unknown	Unknown	Abdominal pain and cloudy PD effluent	Peritoneal fluid culture (+)	Enterocoelia	Rifampicin, doxycycline and amikacin intraperitoneal for 6 weeks	Continued PD
Solak et al. (17)	Turkey	Male	46	Unknown	Contact with sheep and cattle	Abdominal pain, abdominal bloating, and constipation	Negative	Enterocoelia	Rifampicin and ceftriaxone for 45 days	Continued PD
Unal et al. (18), case 1	Turkey	Male	52	Unknown	Unpasteurized cheese consumption	Nausea, vomiting and fever	Peritoneal fluid culture (+)	Enterocoelia	Rifampicin and doxycycline for 6 weeks	Continued PD
Unal et al. (18), case 2	Turkey	Male	38	Unknown	Unpasteurized milk and cheese consumption	Nausea and vomiting	Blood and peritoneal fluid culture (+)	Enterocoelia	Rifampicin and doxycycline for 6 weeks	Continued PD
Alothman et al. (19)	Saudi Arabia	Male	67	Unknown	Unpasteurized milk consumption	Abdominal pain, cloudy dialysate and edema of lower extremity	Peritoneal fluid culture (+)	Enterocoelia	Rifampicin and doxycycline for 2 months	PD catheter removed and shift to hemodialysis
Ozisik et al. (10)	Turkey	Female	39	Yes	Contact with animal	Nausea and severe abdominal pain	Peritoneal fluid culture (+)	Enterocoelia	Rifampicin and doxycycline for 12 weeks	Shift to hemodialysis
Taskapan et al. (20)	Turkey	Male	47	No	Unpasteurized cheese consumption	Fever and cloudy PD effluent	Blood and peritoneal fluid culture (+)	Enterocoelia	Rifampicin and doxycycline for 18 weeks	PD catheter removed and shift to hemodialysis
Present report (2024)	China	Male	56	Yes	Unpasteurized mutton consumption	Fever, abdominal pain and cloudy dialysate	Blood and peritoneal fluid culture (+)	Enterocoelia and abdominal aorta	Rifampicin and doxycycline for 6 weeks	Continued PD

The predominant clinical features noted PD patients with brucellosis peritonitis were abdominal pain (7/10) (10, 14–17, 19, 21)and cloudy dialysate (4/10) (16, 19–21), while our patient exhibited fever, abdominal pain, and cloudy dialysate, which were similar to the manifestations of peritonitis caused by other bacteria. Consequently, depending exclusively on clinical manifestations may result in oversight or misidentification of brucellosis peritonitis. Obtaining a clear infection pathway is crucial for diagnosing brucellosis peritonitis. The majority of cases (8/10) had identifiable sources of brucellosis infection (10, 15, 17–21), such as the consumption of unpasteurized dairy products or direct contact with infected livestock, while a minority (2/10) were unable to determine the source conclusively (14, 16). In this present instance, it is suspected that the transmission of brucellosis occurred through the ingestion of unpasteurized mutton contaminated with Brucella bacteria. What needs to be emphasized is traditional cooking methods, including freezing, smoking, drying, and pickling, are ineffective in eradicating Brucella bacteria unless subjected to prolonged high-temperature sterilization (22, 23). Laboratory detection is essential for the definitive diagnosis of brucellosis peritonitis. The primary method involves confirming the diagnosis through microbial culture from clinical specimens; however, sensitivity may be affected by factors such as sampling time, culture duration, and sample nature (24). Nearly all cases (9/10), including the case of the patient in question, were diagnosed using blood culture or peritoneal dialysis fluid culture to detect Brucella microorganisms. Only one case demonstrated positive results through serum brucella agglutination testing following negative cultures (17). All cases showed infection in the abdomen, but this case was unique as it involved both the abdomen and abdominal aorta. Based on the patient's history and symptoms, we believe the infection may have started in the digestive tract and spread to the peritoneum, eventually reaching the abdominal aorta and inducing inflammatory damage (25).

The therapeutic objective of brucellosis is not only to shorten the duration of symptoms, but also to prevent or reduce brucellosis-related complications and prevent recurrence (26). In 1986, the World Health Organization (WMO) recommended oral doxycycline and rifampin as the primary treatment for focal brucellosis; however, this regimen still exhibits a failure rate of 5–15% (27). Currently, there is no clear consensus on the optimal treatment for PD patients with brucellosis peritonitis. In this study, all cases were treated with a standard regimen of doxycycline combined with rifampin for a duration of 6–18 weeks, but three cases had to switch to minocycline or ceftriaxone because they couldn't tolerate doxycycline (15, 17, 21). The necessity of removing catheters in PD patients with brucellosis peritonitis remains uncertain. Three patients chose to promptly remove their PD catheters during treatment due to abnormal conditions such as positive culture results for Brucella species in the PD catheter or refractory brucellosis peritonitis (15, 19, 20). The majority of PD patients with Brucella infection had no systemic involvement and their symptoms improved with standard treatment, avoiding the need to remove the PD tube. In this case, patient received classic dual therapy for both brucellosis peritonitis and arteritis which resulted in favorable treatment outcomes and prognosis. Therefore, careful consideration should be given to whether removal of PD catheters is necessary after acquiring a Brucella infection based on specific clinical characteristics.

Overall, brucellosis is a rare but treatable cause of peritonitis and arteritis in PD patients, with a good prognosis if diagnosed early and treated promptly. The standard dual therapy is effective for treating PD patients with brucellosis peritonitis and arteritis, but its potential for further improving success rates requires further study. Moreover, It is unclear if PD patients should remove the catheter after brucellosis infection. Therefore, we recommend that decisions should be based on international guidelines and the patient's individual situation.

## Data availability statement

The original contributions presented in the study are included in the article/supplementary material, further inquiries can be directed to the corresponding author.

## Ethics statement

The studies involving humans were approved by Medical Ethics Committee of Shaoxing Second Hospital. The studies were conducted in accordance with the local legislation and institutional requirements. The participants provided their written informed consent to participate in this study. Written informed consent was obtained from the individual(s) for the publication of any potentially identifiable images or data included in this article.

## Author contributions

YH: Writing – original draft, Writing – review & editing. XZ: Writing – original draft, Writing – review & editing. WS: Writing – review & editing, Writing – original draft. MH: Data curation, Supervision, Writing – review & editing. YW: Data curation, Formal analysis, Writing – review & editing.
